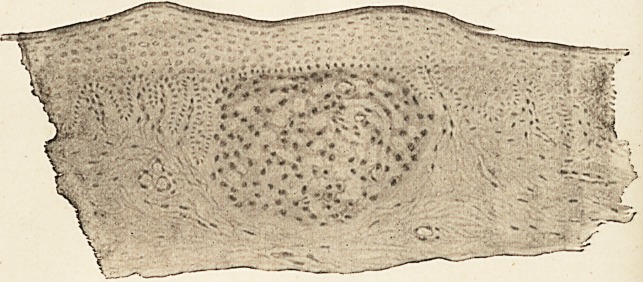# Dermatology

**Published:** 1898-09

**Authors:** Henry Waldo


### DERMATOLOGY.

Dr. Wild,[1] in a paper on some points in the etiology and treatment of **cutaneous tuberculosis,** considers that we are still on the threshold of knowledge in respect to the details of infection, and the variety of morbid appearances which may be due to the operations of the same parasite on different soils and under different conditions of environment; and he thinks this is hardly to be wondered at when we remember that only about fifty years have elapsed since thrush, ringworm, and favus were shown to be due to fungous growth, while the organisms of tubercle, leprosy, actinomycosis, erysipelas, and suppurative processes have only been recognised within the last few years.

Dr. Wild points out that when tubercle bacilli effect an entrance into any tissue we find certain changes taking place in it, which are probably of a protective nature on the part of the organism attacked. These changes consist at first in increased activity of the cells of the invaded tissue, and of the blood-supply going to it; they are included in the term inflammation. The details of the phenomena vary with the tissue affected and with the intensity of the irritating action of the bacilli. Acute tuberculous ulceration occurs usually near the mucous membrane of the orifices of the body, and is due to the direct inoculation of the skin by discharges, such as sputum or fæces, containing

[1] *Med. Chron.*, 1896-97, N.S. vi. 416.

tubercle bacilli. The characteristics of the ulcer are those of a shallow depressed ulcer, little or no induration, slight discharge, and no tendency to heal. These patients are usually in an advanced stage of visceral tuberculosis.

In the group of diseases comprising **verruca necrogenica, tuberculosis verrucosa**, and **lupus verrucosus**, the diseased tissues present bacilli and tubercles, and the discharge also contains bacilli. These forms of disease all agree in presenting red, indurated wart-like growths, sometimes persisting for years. This condition occurs on the hands in *post-mortem* porters, medical men, butchers, and others who handle dead animals affected with tubercle.

Infection, however, occurs in other ways. Dr. Wild reports four cases in women in which the anterior surface of the wrists were affected, through nursing phthisical husbands and washing their linen. No doubt inoculation may take place in many other ways. He believes evidence is accumulating that lupus vulgaris may originate from direct inoculation, as in cracks and fissures on the face of a baby kissed by a phthisical mother; in another case a patch of lupus formed on a tattoo mark which had been moistened by the saliva of a phthisical operator; and he has seen two cases of lupus of the lobule of the ear following perforation for ear-rings.

Bazin's disease or **erythema induratum scrofulosorum** is believed to be tuberculous. The disease consists in chronic, inflammatory, deep-seated nodules, usually in the legs of young women, and boys about the age of puberty, especially those who have much standing at their work. The nodules are painless; sooner or later the skin over them becomes involved, and they break down into circular or irregular-shaped ulcers, having the clean-cut, or punched-out, appearance which is characteristic of syphilitic ulcers. Dr. Wild says they are usually put down as syphilitic lesions. Specific treatment does not cure the lesions of Bazin's disease; rest and cod-liver oil are, however, sufficient, combined with ordinary local measures.

He considers that **lupus erythematosus** bears much the same relation to **lupus vulgaris** that those cases of very chronic phthisis with much fibrous tissue and few apparent tubercles bear to ordinary phthisis. There is an intermediate condition, being that variety of lupus named by Professor Leloir "**lupus vulgaire erythematoïde,**" and which has also been described by other observers as a transformation of lupus erythematosus into lupus vulgaris. The special factors which determine the variety which will result from a local inoculation of tubercle bacilli in the skin may be largely modified (as shown by Dr. Ransome and Professor Delépine) by exposure to light and air, and Dr. Wild thinks it is not unlikely also that different breeds may differ in their pathogenic properties.

Outside the body tubercle bacilli grow most luxuriantly at a temperature of 100° F.; any divergence above or below this point is attended with lessened growth. We should, therefore, expect that tuberculosis of the skin, the surface temperature of which is below that of the internal organs, would be more chronic in its course than the same disease, say, in the lungs, and more especially when the part affected is exposed to light and air, as on the face. Dr. Wild reminds us that tubercle is widely distributed through the animal kingdom, but with a curious partiality: some animals, such as guinea-pigs, rabbits, and monkeys are very susceptible; others, such as dogs, and particularly goats, are almost immune; while men and cows occupy an intermediate position. Man himself also varies much in his susceptibility: some men have no resisting power to the slightest tuberculous infection; while others may live exposed to infection for the whole of a long life without ever falling victims. The susceptibility of the same individual also varies from time to time, as is shown by the frequency with which tuberculosis follows other diseases—*e.g.*, measles or conditions of lowered vitality, by which the power of resisting infection is lessened. The variations may also be due to the exact site of the inoculation in the skin, whether, for example, the bacilli are introduced into the rete Malpighii, into the dermis, or beneath the skin into the subcutaneous tissue.

As Dr. Wild suggests, with these variable factors modifying the course of the disease, we may well comprehend the diverse forms which tuberculosis of the skin assumes. He also points out that lupus and phthisis are both purely local diseases, resulting in local destruction of tissue, the chief difference being that in the case of the lungs the tissue destroyed is essential to life. In both cases also general infection may occur, the firmer texture of the skin, and much less vascularity, accounting for its infrequency in lupus. In both lupus and early phthisis also spontaneous cure may occur, the result in each case being a fibrous cicatrix.

With regard to the treatment of cutaneous tuberculosis, he recognises all forms of the disease as preventible infective lesions, derived from some source which must, if possible, be removed. He recommends the accepted hygienic measures, fresh air (especially sea air), good food, and cod-liver oil. Of drugs he advises creasote or guaiacol, and, perhaps, even goat's serum. The local treatment consists in the destruction of the diseased tissue by mechanical or chemical agents. Precautions should be taken to prevent re-infection by removing the patient from a tuberculous environment and using a[illegible]septic applications.

Dr. Norman Walker, recording[1] a case of **lichen scrophulosorum**, says the alternative names suggested are folliculitis scrophulosorum (Unna), acne cachecticorum (Kaposi), and scrophuloderma papulosum (H. Hebra).

[1] *Scottish M. & S. J.*, 1898, ii. 326.

Sack, H. Hebra, and Jacobi regard it as a form of true tuberculosis of the skin, Jacobi having once found a bacillus which stained like the tubercle bacillus. Dr. Walker says most observers consider that the lesions are probably produced by the toxins of the tubercle bacillus, and he thinks there are strong arguments at least in favour of this as against the other view. He adds that 90 per cent. of the cases show evidence of tuberculosis, usually of the glands; phthisis is comparatively rare, but there is often a history of it in the family. Another argument in favour of the disease being only indirectly tubercular is its course. Almost all the cases get well even if left alone, which, unfortunately, is far from being the case in true tuberculosis.

Dr. Walker considers the disease is usually confined to the trunk, and consists of a number of papules which vary in colour from that of the skin, through yellowish red, up to brownish red. Their size varies, according to different observers, from a pin-head to that of a lentil. They are either conical or flat, they have on their apices a small scale, or occasionally a pustule, and they tend to be arranged in circles or segments of circles—the natural arrangement of the hair follicles. Gradually the colour of the papules fades, and they ultimately disappear. Microscopically, Dr. Walker finds the sections to consist of numerous cells, among which giant cells, epithelioid cells, and round cells are to be distinguished. The giant cell, which is always suspicious, is, however, by no means infrequently found in inflammation of the follicles. As already noted, only once has a somewhat questionable bacillus been found, and Dr. Walker believes that inoculation experiments have not proved successful.

J. I., aged three and a half. The distribution is exceptionally wide. No part of the body escapes, and on the limbs, as is seen in the illustration,[1] the disease is widespread. It also occurs on the face, which is quite exceptional. The spots are only here and there arranged in circles, and so far as one case is evidence it seems to show that the disease is by no means necessarily limited to the follicles. This has already been noted by Sack, who found that one of the nodules which he examined microscopically did not correspond to a follicle. The lesions on the face and leg differed somewhat from the rest, showing a considerable halo, often of a peculiar livid bluish colour, which is also very evident in Bazin's disease, and must be familiar to many in connection with tubercular affections. This tendency of the spots on the legs and the face to differ from the others is noted by Unna. Some of the papules are covered with a yellowish scale, quite a number of them have a pustule at their apex, and some have the smooth top which undoubtedly recalls the lichen papule. The patient is the third in a family of four. His father and mother are both alive and healthy, and there is no history of phthisis on either side. Up to three years ago the boy was perfectly healthy, then he

[1] I have to thank the publishers of the *Scottish Medical and Surgical Journal* for the loan of the block.

began to be listless and disinclined for play. About two months ago the eruption appeared. The spots first appeared on the cheeks, then on the limbs, the trunk being affected last. He takes his food well and sleeps well. The treatment was that recommended by Hebra, namely, cod-liver oil internally and externally.

There is also a case of lichen scrophulosorum recorded by Dr. J. Jackson Clarke,[1] who made a continuous series of sections of the affected skin. One was flat-topped, and is represented in the accompanying figure;[2] another was pointed, but, save for this, resembled the first; a third was placed deeply around the coil of a sweat-gland and did not come near the epidermis.

Dr. Jackson Clarke says that the lesion is definitely limited to one capillary loop and the tissue around it. The endothelial or connective-tissue cells are enlarged, the fibrous tissue is no longer seen, and there is in this lesion no trace of leucocytic infiltration, though doubtless this would be present in the pustular lesions. The formation of the flat-topped papule appears to be due to the stretching or to the thinning of the epidermis covering the summit of a papilla; the stretching being brought about by the inter-papillary processes being thrust apart by the altered condition of the papilla. He adds that the character of the histological change is quite compatible with the lesions being tubercular. The tissue, he says, was unfortunately hardened in such a way (Foa's solution) that it was unsuitable for staining for bacilli.

HENRY WALDO.

### Reviews of Books.

**Ambroise Paré and his Times, 1510—1590.** By STEPHEN PAGET. Pp. xiv., 309. New York: G. P. Putnam's Sons. 1897.

A more interesting personality than that of the subject of this memoir can hardly be found in the whole history of medicine, and we owe a debt of gratitude to Mr. Stephen Paget for giving

[1] *Pediatrics*, 1897, iii. 395. [2] For the loan of the block I am indebted to the courtesy of the publishers of *Pediatrics*.

## Figures and Tables

**Figure f1:**
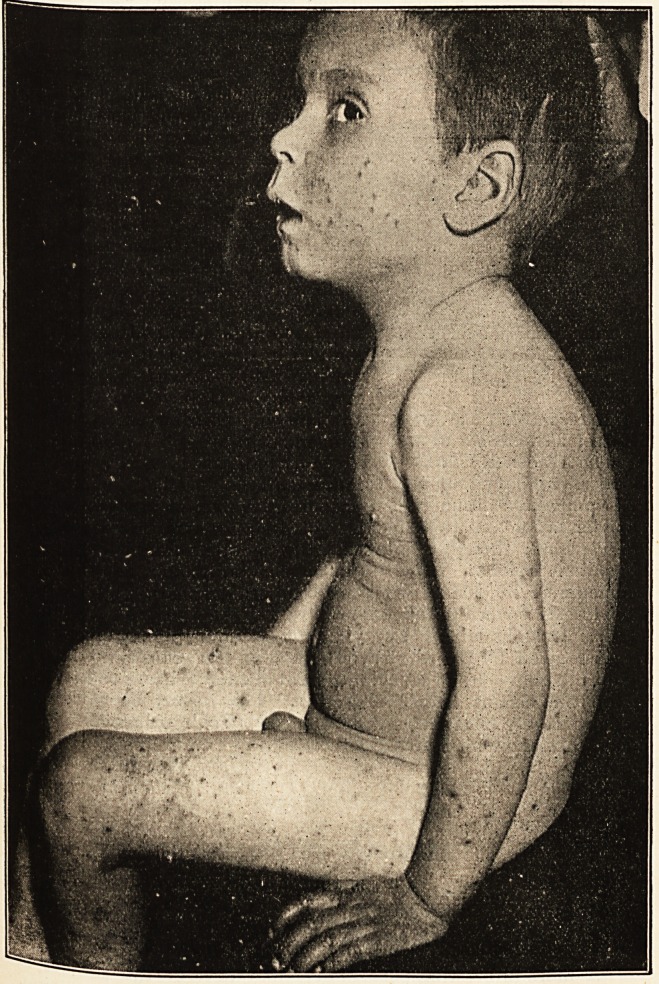


**Figure f2:**